# Bortezomib-dexamethasone as maintenance therapy or early retreatment at biochemical relapse versus observation in relapsed/refractory multiple myeloma patients: a randomized phase II study

**DOI:** 10.1038/s41408-020-0326-1

**Published:** 2020-05-18

**Authors:** Roberto Mina, Angelo Belotti, Maria Teresa Petrucci, Renato Zambello, Andrea Capra, Giacomo Di Lullo, Sonia Ronconi, Norbert Pescosta, Mariella Grasso, Federico Monaco, Claudia Cellini, Marco Gobbi, Stelvio Ballanti, Paolo de Fabritiis, Maria Letizia Mosca-Siez, Monia Marchetti, Anna Marina Liberati, Massimo Offidani, Nicola Giuliani, Roberto Ria, Pellegrino Musto, Alessandra Romano, Pieter Sonneveld, Mario Boccadoro, Alessandra Larocca

**Affiliations:** 1 Myeloma Unit, Division of Hematology, University of Torino, Azienda Ospedaliero-Universitaria Città della Salute e della Scienza di Torino, Torino, Italy; 2 Division of Hematology, Spedali Civili di Brescia, Brescia, Italy; 3 Hematology, Department of Translational and Precision Medicine, Azienda Ospedaliera Policlinico Umberto I, Sapienza University of Rome, Rome, Italy; 4 Padova University School of Medicine, Hematology and Clinical Immunology, Padova, Italy; 5 Istituto Scientifico Romagnolo per lo Studio e la Cura dei Tumori (IRST) IRCCS, Meldola, Italy; 6 Reparto di Ematologia e Centro TMO, Ospedale Centrale, Bolzano, Italy; 7 S.C. Ematologia, Azienda Ospedaliera Santa Croce - Carle, Cuneo, Italy; 8 Dipartimento di Ematologia e Medicina Trasfusionale, Azienda Ospedaliera ‘SS. Antonio e Biagio e Cesare Arrigo’, Alessandria, Italy; 9 U.O.C. EMATOLOGIA, Ospedale Santa Maria delle Croci, Ravenna, Italy; 10 Clinical Hematology, Ospedale Policlinico S. Martino, Istituto di Ricovero e Cura a Carattere Scientifico, Genoa, Italy; 11 Ematologia con TMO, Ospedale Santa Maria della Misericordia di Perugia, Perugia, Italy; 12 Hematology, St. Eugenio Hospital, University Tor Vergata, Rome, Italy; 13 Division of Hematology, Department of Medicine, Ospedale degli Infermi, Biella, Italy; 14 Day Hospital Ematologico, Ospedale Cardinal Massaia, Asti, Italy; 15 Unità di Ematologia, Azienda Sanitaria Ospedaliera ‘Ss. Antonio e Biagio e Cesare Arrigo’, Alessandria, Italy; 16 Università degli Studi di Perugia - Azienda Ospedaliera Santa Maria, Terni, Italy; 17 Clinica di Ematologia, Azienda Ospedaliero-Universitaria Ospedali Riuniti di Ancona, Ancona, Italy; 18 Dipartimento di Medicina e Chirurgia, Università di Parma, Parma, Italy; 19 Internal Medicine “G. Baccelli”, Department of Biomedical Science, University of Bari “Aldo Moro” Medical School, Bari, Italy; 20 Hematology, IRCCS CROB, Rionero in Vulture (Pz), Italy; 21 Unit of Hematology and Stem Cell Transplantation, AOU Policlinico Giovanni XXIII, School of Medicine, Aldo Moro University, Bari, Italy; 22 Division of Hematology, AOU Policlinico-OVE, University of Catania, Catania, Italy; 23 Department of Hematology, Erasmus Medical Center, Rotterdam, Netherlands

**Keywords:** Myeloma, Phase II trials

Disease progression in multiple myeloma (MM) can occur as a biochemical relapse (an increase in monoclonal component without end-organ damage) or as a clinical relapse (a proliferation of plasma cells accompanied by MM-related symptoms). The International Myeloma Working Group recommends that treatment should be initiated in the presence of a clinical relapse or in case of a rapid increase in the monoclonal component. The suitability of early treatment at the occurrence of biochemical relapse is still a matter of debate.

Although continuous therapy prolongs overall survival (OS) as compared to fixed-duration treatment^1–3^, the salvage regimen bortezomib-dexamethasone (Vd, a standard of care and a platform for several triplet regimens) is usually administered for a fixed number of cycles^4,5^.

Here we present the results of a multicenter, randomized phase II study aiming at evaluating efficacy and safety of either Vd maintenance or Vd retreatment at the occurrence of biochemical relapse as compared to standard observation in MM patients who received a bortezomib-based salvage regimen at relapse.

Patient eligibility, study design and statistical analysis are summarized in the Supplementary Appendix. Briefly, patients with relapsed/refractory (RR)MM (1–3 previous therapies) treated with a bortezomib-based regimen as last line of therapy without evidence of progression were randomized to: continuous treatment with subcutaneous bortezomib (1.3 mg/m^2^; days 1,15) and oral dexamethasone (20 mg; days 1, 2, 15, 16) every 28 days until progression (arm A); observation until clinical relapse as per standard of care (arm B); six 28-day cycles of subcutaneous bortezomib (1.3 mg/m^2^; days 1, 8, 15, 22) and oral dexamethasone (40 mg; days 1, 8, 15, 22) at the occurrence of biochemical relapse. The primary objective was to determine the time to progression (TTP), calculated as either the time from enrollment to biochemical relapse (TTBR) or the time from enrollment to clinical relapse (TTCR). This trial was conducted in accordance with the Declaration of Helsinki and the principles of Good Clinical Practice and was registered at ClinicaTrials.gov (NCT01913730).

We analyzed 58 patients (median age: 70 years) enrolled from 21 Nov 2013 to 16 Mar 2017 and randomized to the three arms (*A* = 15, *B* = 20, *C* = 23, Supplementary Fig. S1; see Supplementary Table S1 for patient characteristics). On 22 Jul 2015, the protocol was amended: the arm A (Vd maintenance) was closed due to low speed of enrollment and the sample size was reduced.

In arm A, all patients (15/15) started Vd maintenance (median number of 11 maintenance cycles). In arm B, 18 patients (90%) experienced a biochemical relapse, in all cases followed by a clinical relapse, and started a subsequent line of therapy. In arm C, 21 patients had a biochemical relapse: 17 started therapy with Vd (median number of cycles: 6), while 4 patients did not (2 concomitant clinical relapses; 1 death; 1 consent withdrawal); 19 patients (83%) had a subsequent clinical relapse.

In arm A, the best response with Vd maintenance was at least a complete response (≥CR) in 20% of patients, very good partial response (VGPR) in 13% and PR in 20% (Supplementary Table S2); 33% of patients improved by at least 1 category the response achieved with the previous therapy.

In arm C, Vd after the occurrence of a biochemical relapse resulted into an overall response rate (ORR) of 30% (PR, 6%; VGPR, 24%), with 82% of patients achieving at least a stable disease (SD), while 18% of patients progressed while on therapy (PD).

After a median follow-up of 41 months, TTBR was longer in patients receiving Vd maintenance (arm A, 18.2 months) than in patients who were observed until the occurrence of biochemical relapse (arm B, 4.9 months; arm C, 8.4 months; Fig. 1). TTCR was longer in patients treated with Vd maintenance (arm A, 22.1 months) or Vd at biochemical relapse (arm C, 20.3 months) than in patients under observation only (arm B, 9.5 months), being similar in the two experimental arms (A, C).

**Fig. 1:**
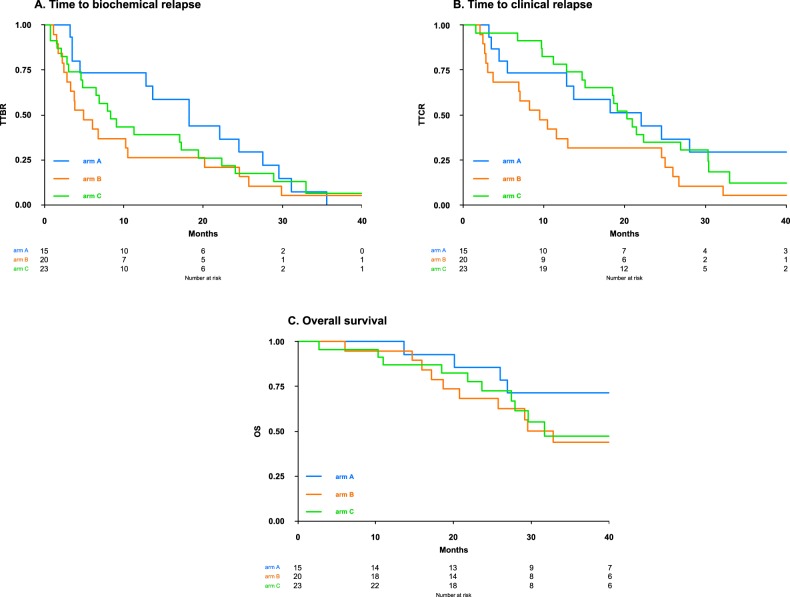
Survival outcomes. Time to biochemical relapse (**a**), time to clinical relapse (**b**) and overall survival (**c**) in the examined population. TTBR time to biochemical relapse, TTCR time to clinical relapse, OS overall survival, ARM A bortezomib and dexamethasone until progression, ARM B observation until clinical relapse, ARM C early retreatment at biochemical relapse with bortezomib and dexamethasone.

The median second progression-free survival (2nd PFS) was 20.5, 11.8 and 8.2 months in arms A, B and C, respectively.

A longer median OS was reported with Vd maintenance (arm A, 45.1 months), as compared to observation (arm B, 32.8 months) or Vd at biochemical relapse (arm C, 31.7 months), although this difference was not statistically significant.

Treatment was well tolerated, with limited grade 3–4, treatment-related adverse events (AEs; arm A 20%, arm B, 6%). Peripheral neuropathy (PN), mainly of grade 1–2, occurred in 20 and 12% of patients in arms A and C, respectively. Treatment discontinuation due to AEs was reported in 20 and 6% of patients in arms A and C, respectively (Table 1).

**Table 1:** Treatment-related adverse events during the study treatment.

Adverse events	Arm A (*n* = 15) All grades (%)	Arm A (*n* = 15) Grade ≥ 3 (%)	Arm C (*n* = 17) All grades (%)	Arm C (*n* = 17) Grade ≥ 3 (%)
**Hematologic**				
At least 1 event	13	0	47	0
Anemia	7	0	29	0
Neutropenia	7	0	12	0
Thrombocytopenia	7	0	12	0
**Non-hematologic**				
At least 1 event	33	20	53	6
Cardiologic	0	0	6	0
Vascular	7	7	12	0
Infection	13	7	24	6
Nervous	20	7	18	0
*Peripheral neuropathy*	20	7	12	0
Gastrointestinal	13	0	6	0
**Discontinuation due to adverse events**	20	–	6	–

Continuous treatment is a standard approach in newly diagnosed (ND)MM and RRMM patients. In NDMM, continuous lenalidomide is a standard of care^1,6^. Maintenance therapy with the oral proteasome inhibitor ixazomib proved to be an effective strategy in delaying disease progression after ASCT^2^. Although bortezomib maintenance can extend PFS and OS in both transplant-eligible and -ineligible patients^3,7^, bortezomib is usually administered for a limited number of cycles, primarily due to the risk of PN and the inconvenience for patients attending hospital for subcutaneous administration^6–10^.

We hypothesized that the use of continuous Vd, in patients sensitive to a bortezomib-based salvage regimen as last line of therapy, would maintain, and even deepen, the previously achieved depth of response, ultimately delaying disease progression in comparison with observation. In our study, the administration of Vd maintenance delayed by approximately 1 year TTBR (18.2 vs. 4.9 months) and TTCR (22.1 vs. 9.5 months), as compared to the control arm, in which patients were observed until the occurrence of clinical progression as per standard of care. Importantly, maintenance therapy did not negatively impact the efficacy of the subsequent lines of therapy, as shown by the longer median 2nd PFS (20.5 months) in the maintenance arm compared to that in the observation arm (11.8 months; Supplementary Table S3)^1,2,7^. Vd maintenance was well tolerated, with a limited rate of PN (12% all grades; 6% grades 3–4). These findings are consistent with a phase 2 study that tested Vd maintenance in RRMM, reporting an ORR of 34.5% and a median TTP of 17 months^11^. These results are of interest in the context of novel bortezomib-based combinations, such as daratumumab-Vd, in which bortezomib is administered only for 8 cycles^5^.

Salvage therapy is currently recommended in case of clinical relapse. However, this strategy is in contrast with the current definition of MM, which includes not only signs and symptoms of a clinically overt MM, but also markers predictive of an early imminent progression, prompting a therapeutic intervention in asymptomatic patients to prevent the development of MM-related comorbidities^12^. In a Spanish trial, the median interval between biochemical and clinical relapse was 5.1 months^13^. Furthermore, there is evidence that biochemical relapse precedes the onset of a clinical relapse by several months^13^, and that early retreatment at biochemical relapse, rather than at clinical relapse, can delay disease progression and the onset of significant myeloma-related comorbidities, thus improving patients’ quality of life^14^.

To our knowledge, this is the first randomized study that prospectively evaluated the efficacy and safety of early treatment at biochemical relapse in MM.

Early intervention with Vd induced disease stability or better in 82% of the treated patients and delayed clinical progression by ~11 months, as compared to observation until clinical relapse (20.3 vs. 9.5 months). These results confirmed the findings of previous studies. In the REBOUND trial, retreatment with a bortezomib-based regimen induced an ORR of 71% and a median PFS of 15 months. Retreatment with Vd was well tolerated, with a limited rate of PN^15^. The evidence generated by our study represents a proof-of-concept, suggesting that early retreatment in MM patients who previously benefited from a bortezomib-based therapy—with the aim of preventing MM-related anemia, bone lesions, renal failure and hypercalcemia – is feasible and effective.

Limitations of this study were the long enrollment time and the small sample size of enrolled patients, which limited the statistical significance of the comparisons. Also, the small sample size precluded subgroup analyses to understand whether a specific subset of patients, as those with a suboptimal response (<CR), could benefit more from continuous therapy or early retreatment with the same drug than those who had achieved CR (or vice versa). Despite these limitations, we were able to capture a clinically meaningful difference in TTBR and TTCR between the experimental arms (A, maintenance, and C, retreatment) and the control arm (observation, B). The study was specifically designed to compare each one of the experimental arms to the control arm, but not to one another. Consequently, we are unable to draw definite conclusions on the best bortezomib-based strategy to delay clinical relapse, whether a continuous, gentler approach after the induction phase or a close observation followed by early bortezomib retreatment at the occurrence of biochemical relapse, in order to allow patients a treatment-free period.

In conclusion, we demonstrated that, in RRMM treated with a bortezomib-based salvage therapy, maintenance therapy with Vd or early retreatment with Vd at the occurrence of biochemical relapse were safe and effective strategies to delay clinical progression without negatively affecting the efficacy of subsequent lines of therapy.

## Supplementary information

Supplementary Appendix

Reporting checklist

CONSORT Checklist
