# Supplementary material for: Bortezomib-dexamethasone as maintenance therapy or early retreatment at biochemical relapse versus observation in relapsed/refractory multiple myeloma patients: a randomized phase II study
Source: Blood Cancer J. 2020 May 18;10(5):58. doi: 10.1038/s41408-020-0326-1 (PMC7234990; doi:10.1038/s41408-020-0326-1)
Supplement: Supplementary file 1 — Supplementary Appendix [file 41408_2020_326_MOESM1_ESM.pdf]

Bortezomib-Dexamethasone as Maintenance Therapy or Early Retreatment at Biochemical Relapse Versus Observation in Relapsed/Refractory Multiple Myeloma Patients: A Randomized Phase II Study

Supplementary Appendix

- **Additional methods**
  - Patients
  - Study design and treatment
  - Assessment
  - Statistical analysis
  - Role of the funding source

- **Supplementary figures and tables**
  - Table S1. Baseline patient characteristics in the ITT population
  - Table S2. Best overall response
  - Table S3. Subsequent lines of therapy
  - Figure S1. Study flow

- **Additional References**

Additional methods

Patients

Patients with relapsed and/or refractory multiple myeloma (RRMM), who were ≥18 years old and who had received 1 to 3 prior lines of therapy were included in the study. Patients had to have measurable disease, defined as any quantifiable serum monoclonal protein value and, where applicable, urine light-chain excretion >200 mg/24 hours or, in case of non-secretory MM, >30% of plasma cells in the bone marrow and at least 1 plasmacytoma >2 cm as determined by clinical or radiological examination. Additionally, patients had to have received at least 4 cycles of a bortezomib-containing regimen as their last line of therapy (no later than 45 days prior to enrollment) without the evidence of disease progression. Further eligibility criteria included: Karnofsky performance status ≥60%, adequate bone marrow (hemoglobin level ≥8 gr/dl; platelet count ≥80.000x10^9/L, absolute neutrophil count (ANC) 1x10^9/l) and liver function (AST and ALT <2.5 the upper limit of normal [ULN], total bilirubin <1.5 ULN) and a creatinine clearance ≥20 ml/min. Patients were excluded from the study if they had grade ≥2 peripheral neuropathy and uncontrolled or severe cardiovascular disease, including myocardial infarction within 6 months of enrolment, New York Heart Association (NYHA) Class III or IV heart failure, uncontrolled angina, clinically significant pericardial disease, or cardiac amyloidosis which prevented the use of study drug, or uncontrolled diabetes which prevented the use of high-dose dexamethasone, or infarction, or unstable angina ≤4 months before. All patients gave written informed consent to participate in the study, which had been approved by the institutional ethics committees and review boards at the study sites. The study was conducted in accordance with the Declaration of Helsinki and the principles of Good Clinical Practice. This trial was registered at Clinicatrials.gov as NCT01913730.

Study design and treatment

This was a multicenter, randomized, open label phase II study to determine the superiority of either continuous treatment with bortezomib-dexamethasone (Vd; *arm A*) or retreatment at the occurrence of biochemical relapse, defined as an increase of 25% from the lowest response value in serum M-component (absolute increase >0.5 g/dL) and/or urine M-component (absolute increase >200 mg/24 h) (*arm C*), versus observation as per standard of care (*arm B*). Clinical relapse was defined as the occurrence of any myeloma-related organ damage, including anemia, hypercalcemia, increased serum creatinine or the appearance of a new bone lesion or extramedullary plasmacytoma. This trial consists of a double study aiming to demonstrate the superiority of the 2 experimental arms, each compared with the control arm (arm B, observation), without a comparison between the 2 experimental arms. Patients were randomized in a 1:1:1 ratio to the 3 arms. The primary objective of the study was to determine the time to progression (TTP), calculated as either the time from enrollment to biochemical relapse (TTBR) or the time from enrollment to clinical relapse (TTCR). Secondary objectives were progression-free survival (PFS), second PFS (2nd PFS), overall survival (OS), overall response rate (ORR) and safety. In arm A, patients received continuous treatment with subcutaneous bortezomib at the dose of 1.3 mg/m^2 on days 1 and 15 and oral dexamethasone at the dose of 20 mg on days 1, 2, 15 and 16 every 28 days until progression; in arm B, patients received no treatment until the occurrence of clinical relapse as per standard of care; in arm C patients were treated at the occurrence of biochemical relapse with six 28-day cycles of subcutaneous bortezomib at the dose of 1.3 mg/m^2 on days 1, 8, 15 and 22 and oral dexamethasone at the dose of 40 mg on days 1, 8, 15 and 22; treatment was to be repeated at each biochemical relapse until the occurrence of clinical relapse.

Assessment

All adverse events (AEs) were assessed during each cycle and graded according to the national Cancer Terminology Criteria for Adverse Events (version 3.0).[1]

Responses were recorded at the beginning of every cycle, according to the International Myeloma Working Group (IMWG) criteria;[2] near-complete response (nCR) was defined as evidence of monoclonal component at immunofixation with less than 5% of monoclonal plasma cells in the bone marrow.

Fluorescence in situ hybridization (FISH) was used to detect t(4;14), t(11;14), t(14;16) and del(17p).

Statistical analysis

The sample size was based on the comparison between the experimental arms (A and C) and the control arm (B), in order to demonstrate the superiority of the experimental arms compared with the control arm. The total number of 186 patients, 62 per arm, was planned. On July 22, 2015, the protocol was amended: the arm A (Vd maintenance) was closed due to low speed of enrollment and the sample simple size was reduced. The final sample size calculation (without arm A as Vd maintenance) was based on one-step Fleming design. At 12 months, the TTCR rate for bortezomib-containing regimens in RRMM was approximately 20%, which was the expected time to progression (TTP) for the control arm (arm B). Assuming that the experimental treatment (arm C) could increase the TTP rate to 45%, the total number of patients needed for this study was equal to 38 (19/arm) with one-sided alpha 0.05 and power (1-β) 0.80. Assuming 20% of patients were lost to follow-up, an adequate sample size was thus 46 patients (23/arm).

Time-to-event endpoints were determined using the intention-to-treat principle by using the Kaplan–Meier product limit method, with median and 95% confidence interval (CI). TTBR was measured from the date of randomization to the date of first observation of biochemical relapse or death from progression. Subjects who did not progress were censored at the cut-off date. TTCR was measured from the date of randomization to the date of first observation of clinical progression or death from progression. 2nd PFS was measured from the date of the start of the subsequent line of treatment to the date of observation of progression after this line of therapy or death to any cause as an event. OS was defined as the time between randomization and death. Response rates and safety were analyzed in patients who received ≥1 dose of study treatment.

Statistical analyses were performed by using R (version 3.5.1). The data cut-off was March 30, 2015.

Role of the funding source

This study was sponsored by the HOVON foundation, co-sponsored in Italy by the Fondazione Neoplasie Sangue (FO.NE.SA.) ONLUS, and supported by funding from Janssen. The funders had no role in the collection, analysis or interpretation of data. All authors had full access to all data in the study and had final responsibility for the decision to submit for publication.

Table S1. Baseline patient characteristics in the ITT population

	All patients N=58 (%)	Arm A N=15 (%)	Arm B N=20 (%)	Arm C N=23 (%)
Age – median (range)	70 (48-89)	72 (50-89)	68 (48-85)	72 (63-83)
ECOG PS				
0	36 (62)	11 (73)	11 (55)	14 (61)
1	20 (34)	3 (20)	8 (40)	9 (39)
2	2 (3)	1 (7)	1 (5)	-
ISS				
1	40 (69)	10 (67)	15 (75)	15 (65)
2	11 (19)	3 (20)	2 (10)	6 (26)
3	7 (12)	2 (13)	3 (15)	2 (9)
Cytogenetic risk				
high	5 (28)	-	-	5 (45)
standard	13 (72)	2 (100)	5 (100)	6 (55)
missing	40	13	15	12
Previous lines of therapy N (%)				
1	4 (7)	-	4 (20)	-
2	31 (53)	8 (53)	12 (60)	11 (48)
3	23 (39)	7 (47)	4 (20)	12 (52)
Previous lines with IMiDs – N (%)				
lenalidomide	22 (37)	4 (25)	6 (30)	12 (52)
thalidomide	5 (8)	3 (19)	1 (5)	1 (4)
Previous ASCT– N (%)	21 (36)	6 (37)	5 (25)	10 (43)
Prior bortezomib-based regimen*				
Vd	43(74)	9 (60)	15 (75)	19 (83)
VMP	3 (5)	1(7)	2 (10)	-
VCD	7 (12)	2(13)	1 (5)	4 (17)
PAD	2 (3)	1(7)	1 (5)	-
VTd	1(2)	1(7)	-	-
BVd	2(3)	1(7)	1 (5)	-
Best response to the last line of therapy				
CR	4 (7)	1 (7)	2 (10)	1 (4)
VGPR	16 (28)	5 (33)	5 (25)	6 (26)
PR	21 (36)	6 (40)	6 (30)	9 (39)
SD	17 (29)	3 (20)	7 (35)	7 (30)
ORR	41 (71)	13 (80)	13 (65)	16 (70)

Abbreviations. ITT, intention-to-treat; N, number; IQR, interquartile range; ISS, International Staging System stage; ECOG PS, Eastern Cooperative Oncology Group Performance Status; IMiDs, immunomodulatory drugs; ASCT, autologous stem-cell transplantation; Vd, bortezomib-dexamethasone; VMP, bortezomib-melphalan-prednisone; VCD, bortezomib-cyclophosphamide-dexamethasone: PAD, bortezomib-doxorubicin-dexamethasone; VTD, bortezomib-thalidomide-dexamethasone; BVd, bendamustine-bortezomib-dexamethasone; CR, complete response; VGPR, very good partial response; PR, partial response; SD, stable disease; ORR, overall response rate.

*It refers to the last bortezomib-based regimen patients received immediately prior to the enrollment.

Table S2. Best overall response

Best response	Arm A (N=15)	Arm C (N=17)
sCR	2 (13)	-
CR	2 (7)	-
VGPR	2 (13)	4 (24)
PR	3 (20)	1 (6)
SD	1 (7)	9 (53)
PD	5 (33)	3 (18)
NE	1 (7)	-

Abbreviations. N, number; CR, complete response; sCR, stringent CR; VGPR, very good partial response; PR, partial response; SD, stable disease; PD, progressive disease; NE, not evaluable.

Table S3. Subsequent lines of therapy

	All N=48 (%)	Arm A N=11 (%)	Arm B N=18 (%)	Arm C N=19 (%)
**Subsequent therapy: Yes**	41 (85)	7 (64)	18 (100)	16 (84)
Bortezomib-containing regimen	2 (4)	-	2 (11)	-
Lenalidomide-containing regimen	10 (21)	2 (18)	5 (28)	3 (16)
Daratumumab-containing regimen	1 (2)	-	-	1 (5)
Conventional chemotherapy	1 (2)	-	1 (6)	-
Other	27 (56)	5 (45)	10 (56)	12 (63)
**Subsequent therapy: No**	7 (15)	4 (36)	-	3 (16)

Figure S1. Study flow

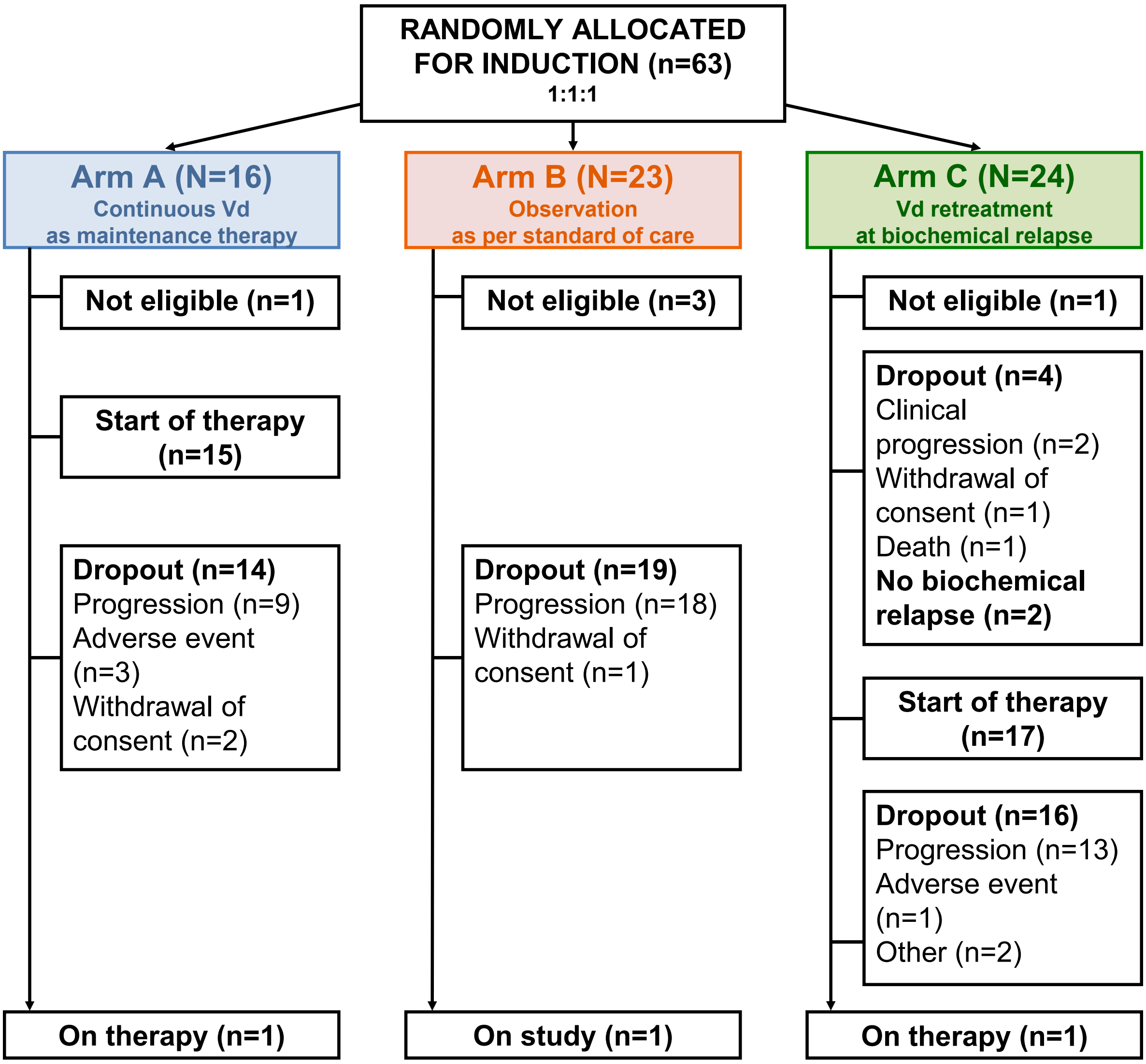

Vd, bortezomib-dexamethasone; *ARM A*: bortezomib (1.3 mg/m^2, days 1,15) and dexamethasone (20 mg, days 1,2,15,16) until progression; *ARM B*: observation until clinical relapse as per standard of care; *ARM C*: early retreatment at biochemical relapse with six 4-week cycles of bortezomib (1.3 mg/m^2 on days 1,8,15,22) and weekly dexamethasone (40 mg).

Additional References

1. National Cancer Institute (US) - Cancer Therapy Evaluation Program. Common Terminology Criteria for Adverse Events (CTCAE) v3.0. https://ctep.cancer.gov/protocoldevelopment/electronic_applications/docs/ctcaev3.pdf (accessed 27 Jan2020).

2. Kumar S *et al*. International Myeloma Working Group consensus criteria for response and minimal residual disease assessment in multiple myeloma. Lancet Oncol 2016; 17: e328–e346.
